# Massive retroperitoneal uterine fibroid causing life-threatening pulmonary embolism: A case report

**DOI:** 10.1016/j.crwh.2026.e00807

**Published:** 2026-04-01

**Authors:** Tomohito Kobiyama, Eriko Iito, Mao Sekimata, Naoki Abe, Sachino Kira, Sotaro Hayashi, Masamitsu Kurakazu, Lifa Lee, Satoshi Nishiyama, Hiroshi Tsujioka

**Affiliations:** Department of Obstetrics and Gynecology, Aso Iizuka Hospital, Iizuka, Japan

**Keywords:** Uterine fibroids, Retroperitoneal cavity, Immobility, Pulmonary embolism

## Abstract

Uterine fibroids are common in women of reproductive age and frequently remain asymptomatic. However, depending on their location, they may lead to severe symptoms or complications. This report describes a case in which a large uterine fibroid extending into the retroperitoneal cavity obstructed venous return. In addition, pelvic nerve compression resulted in pain-induced immobility, which precipitated sudden and severe thromboembolism. The patient required emergency thrombectomy via thoracotomy, followed by definitive hysterectomy. Anticoagulation therapy was continued postoperatively; following a regression of the thrombus, the treatment was switched to oral anticoagulants. The patient was eventually discharged with an uneventful clinical course and no complications. This case underscores the critical importance of appropriately timing surgical management for uterine fibroids, with careful consideration of tumor location and associated symptoms, to prevent potentially fatal outcomes.

## Highlights

• A retroperitoneal uterine fibroid caused venous obstruction and immobility.
• Pain-related immobility triggered deep vein thrombosis and pulmonary embolism.
• The massive pulmonary embolism required emergency thrombectomy and support.
• Early surgery should be considered for symptomatic retroperitoneal fibroids.

## 1 Introduction

Uterine fibroids are a common benign disease in women of reproductive age, with a prevalence of up to 70% [1], [2]. Most are asymptomatic and discovered incidentally during clinical examination or ultrasonography [3]. Common symptoms of uterine fibroids include abnormal uterine bleeding, pelvic pain, pressure-related symptoms, and infertility [4]. Among the different types, subserosal fibroids tend to be less symptomatic than intramural or submucosal fibroids. Because subserosal fibroids often grow into the expansive peritoneal cavity, it is not uncommon for them to remain asymptomatic even when they reach a large size. The case reported, however, is notable because a subserosal fibroid grew into the confined space of the retroperitoneal cavity, resulting in a rare constellation of symptoms, including venous return obstruction and pain due to compression of pelvic nerves. The immobility resulting from this pain may have further to the development of thromboembolism.

## 2 Case Presentation

A 49-year-old nulligravid Japanese woman with a known history of uterine leiomyoma presented to an orthopedic clinic with progressively worsening left lower limb and lower back pain. An abdominal X-ray revealed a calcified pelvic mass, prompting referral to obstetrics and gynecology.

**Fig. 1:**
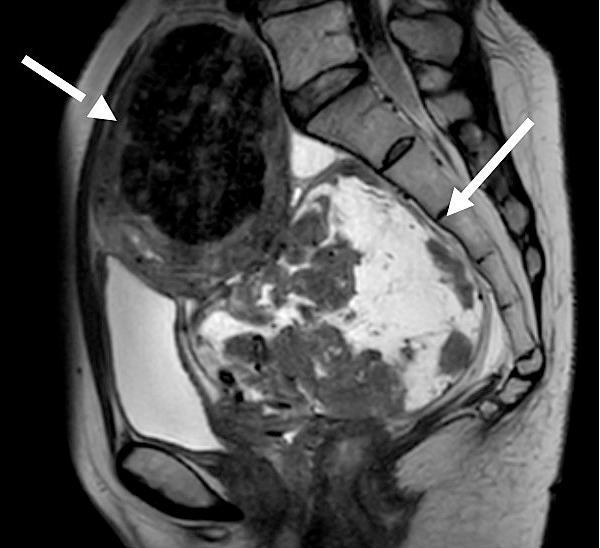
Sagittal magnetic resonance imaging revealed a 58-mm fundal leiomyoma (broken arrow) and a 120 mm × 70 mm retroperitoneal mass (arrow).

**Fig. 2:**
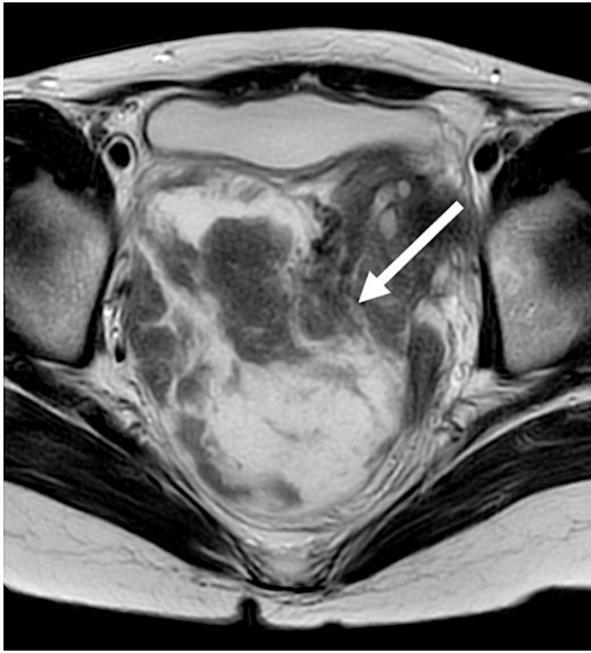
Axial magnetic resonance imaging revealed a 120 mm × 70 mm mass extending extensively into the retroperitoneum (arrow).

Transvaginal ultrasonography identified a leiomyoma, 58 mm × 58 mm, in the uterine fundus and a distinct hypervascular retroperitoneal mass, 90 mm × 84 mm. Magnetic resonance imaging confirmed the 58-mm fundal leiomyoma and measured the retroperitoneal mass at 120 mm × 70 mm, suggesting a degenerating subserosal leiomyoma (Fig. 1, Fig. 2). The patient was hospitalized because of severe pain-induced immobility. Initial contrast-enhanced computed tomography and lower extremity venous ultrasonography showed no evidence of metastasis or venous thrombosis. D-dimer levels were mildly elevated (0.9 μg/mL). The left leg pain was clinically attributed to sciatic nerve compression by the presumed tumor. An abdominal simple total hysterectomy with bilateral salpingectomy was scheduled for the seventh day of hospitalization.

**Fig. 3:**
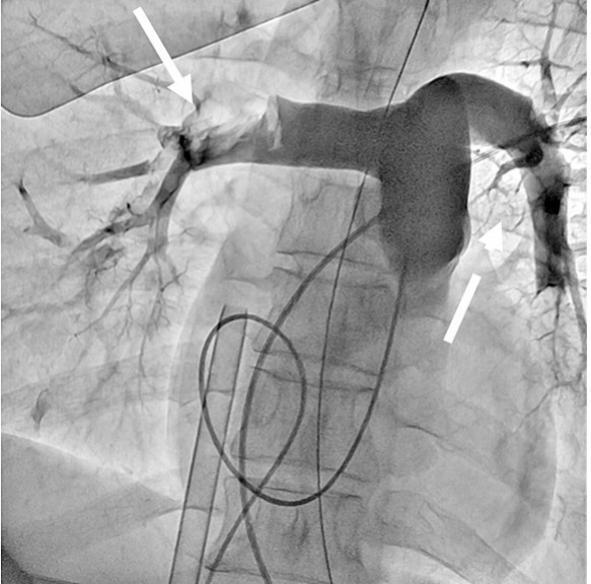
Catheter-based pulmonary angiography confirmed massive bilateral PE (arrow and broken arrow).

On the fourth day of hospitalization, the patient suddenly experienced cardiopulmonary arrest. Immediate cardiopulmonary resuscitation resulted in cycles of pulseless electrical activity followed by the return of spontaneous circulation. Echocardiography revealed right-sided cardiac dilation, strongly suggesting acute pulmonary embolism (PE). Emergency catheter-based pulmonary angiography confirmed massive bilateral PE (Fig. 3). Given the patient's ongoing hemodynamic collapse, veno-arterial extracorporeal membrane oxygenation was immediately initiated, followed by catheter-directed pulmonary artery thrombus fragmentation.

However, persistent thrombotic occlusions in the right main and left lower lobe pulmonary arteries, together with refractory hemodynamic instability, necessitated an emergency open pulmonary embolectomy. The was successfully performed under cardiopulmonary bypass with induced cardiac arrest, allowing removal of the remaining thrombi and resulting in marked hemodynamic improvement. The patient was successfully weaned from bypass without intraoperative complications and transferred to the intensive care unit.

On the fifth day, heparin anticoagulation was resumed, and an inferior vena cava filter was temporarily placed to mitigate the risk of further emboli. On the sixth day, following bilateral ureteral stent placement, an abdominal simple total hysterectomy with bilateral salpingo-oophorectomy was performed. Intraoperatively, two subserosal leiomyomas were identified: an 8-cm mass at the uterine fundus and an 11.5-cm mass arising from the cervix and extending into the retroperitoneum. The surgery was uneventful, and the ureteral stents were subsequently removed. Heparin anticoagulation was resumed on the seventh day.

On the eighth day, follow-up lower extremity venous ultrasonography identified 7-cm and 5-cm thrombi in the left and right soleal veins, respectively. Because the deep vein thrombosis (DVT) was localized below the knee, anticoagulation was continued and the inferior vena cava filter was removed. On the ninth day, anticoagulation was transitioned from intravenous heparin to oral edoxaban. The patient was discharged from the intensive care unit as her condition stabilized.

By the 17th day, ultrasonography showed complete resolution of the right soleal vein thrombus and reduction of the left soleal vein thrombus to 6 cm. Contrast-enhanced computed tomography on the 29th day confirmed resolution of all pulmonary artery thrombi, with further reduction of the left soleal vein thrombus to 3.8 cm. The patient was discharged home on the 35th day with no apparent sequelae. Oral anticoagulation was discontinued after 3 months. Subsequent investigation revealed no underlying abnormalities in coagulation factors.

## 3 Discussion

Uterine fibroids are the most common benign smooth muscle tumors of the uterus, with reported incidence rates of up to 70% [1], [2]. Although common, PE secondary to DVT associated with uterine leiomyoma is rare, with only a few cases documented in the English-language literature over the past decade. Given that the annual incidence of acute PE ranges from 39 to 115 per 100,000 individuals and that hemodynamically unstable PE is acutely life-threatening [5], recognition and mitigation of this rare condition in patients with leiomyoma are critical. Approximately 90% of PEs originate from thrombi in the deep veins of the lower limbs or the pelvic veins [6], underscoring that the pathophysiology of PE shares the same risk factors as DVT.

The development of venous thromboembolism (VTE) is classically explained by Virchow's triad: endothelial injury, venous stasis, and hypercoagulability [7]. In the present case, the underlying mechanism appears to have been driven primarily by venous stasis, exacerbated by immobilization.

The patient's large (11.5 cm) leiomyoma, originating from the uterine cervix and extending extensively into the retroperitoneal cavity, acted as a physical obstruction. This mass likely exerted significant extrinsic compression on the bilateral common iliac veins and adjacent venous structures. Although the lesion was pathologically confirmed as a leiomyoma, its unusual growth pattern raised the possibility of rare variants, such as cotyledonoid dissecting leiomyoma. Regardless of the exact subtype, its retroperitoneal extension directly predisposed the patient to impaired venous return and subsequent stasis.

Following hospitalization, the patient developed severe lower extremity and lumbar pain attributed to compression of surrounding pelvic nerves, including the sciatic nerve. This pain resulted in 5 consecutive days of profound immobility, leading up to the catastrophic PE event. Prolonged immobilization is a well-established and powerful independent risk factor for VTE and is incorporated into the Wells criteria when immobilization exceeds 3 days [7]. In this case, tumor-induced nerve compression caused functional immobility, further amplifying venous stasis already present due to mechanical venous compression.

Despite initial imaging and laboratory evaluations showing no evidence of DVT on admission (D-dimer 0.9 μg/mL, a value often considered below the clinically significant threshold for VTE), the combined effects of mechanical venous compression and pain-induced immobilization likely precipitated DVT, followed by massive PE. Moreover, the absence of detectable DVT at initial evaluation meant that prophylactic anticoagulation was not initiated during hospitalization—a decision consistent with standard protocols but ultimately insufficient in this high-risk setting.

As this case illustrates, when a uterine leiomyoma demonstrates extensive retroperitoneal or pelvic growth in combination with clinically significant pain-induced immobility, the patient's risk of DVT may be substantially higher than typically anticipated. In such cases, early surgical intervention should be considered, when feasible, as a primary strategy to reduce life-threatening thromboembolic risk. For patients awaiting elective surgery, a heightened index of suspicion is warranted, and prophylactic anticoagulation should be strongly considered in the interim.

## 4 Conclusion

This case highlights that when a uterine leiomyoma exhibits extensive retroperitoneal extension in combination with compounding thrombotic risk factors, such as immobilization, the potential for catastrophic PE must be proactively recognized. In such situations, early definitive surgical intervention should be strongly considered to minimize the risk of severe thromboembolic complications.

## Contributors

All authors contributed to patient care, conception of the case report, drafting the manuscript and revising the article critically for important intellectual content.

All authors approved the final submitted manuscript.

## Patient consent

Written informed consent was obtained from the patient for publication of this case report and accompanying images.

## Provenance and peer review

This article was not commissioned and was peer reviewed.

## Funding

This work did not receive any specific grant from funding agencies in the public, commercial, or not-for-profit sectors.

## Declaration of competing interest

The authors declare that they have no competing interest regarding the publication of this case report.
